# Antibiotic susceptibility in commonly isolated pathogens from urinary tract infection in a cohort of subjects from low socioeconomic strata

**DOI:** 10.12669/pjms.332.11569

**Published:** 2017

**Authors:** Saera Suhail Kidwai, Ayesha Nageen, Samina Ghaznavi, Farhat Bashir, Jamal Ara

**Affiliations:** 1 Saera Suhail Kidwai, Associate Professor, United Medical and Dental College – Creek General Hospital, Karachi, Pakistan; 2 Ayesha Nageen, Assistant Professor, United Medical and Dental College – Creek General Hospital, Karachi, Pakistan; 3 Samina Ghaznavi, Assistant Professor, Liaquat National Hospital and Medical College, Karachi, Pakistan; 4 Farhat Bashir, Associate Professor, United Medical and Dental College – Creek General Hospital, Karachi, Pakistan; 5 Jamal Ara, Professor and Head, Department of Medicine, United Medical and Dental College – Creek General Hospital, Karachi, Pakistan

**Keywords:** Antibiotic resistance, Culture and sensitivity, Urinary tract infection

## Abstract

**Background and Objective:**

Urinary tract infection is one of the commonest infections seen in clinical practice. Lack of compliance and unjustified antibiotic prescriptions has resulted in bacterial resistance and is proving as a major challenge in the management of these infections. Our aim was to identify the sensitivity pattern of commonly used antibiotics against urinary tract infections so as to suggest an improvised line of action against bacteria causing urinary tract infections’.

**Method:**

This was a hospital based cross sectional study extended over a period of four months. Patients were recruited from outpatients department of a tertiary care hospital in an industrial area of Karachi. Adult patients with symptomatic and documented UTI in urine detailed report (pus cells >10) were enrolled after informed consent. A clean catch midstream urine was collected for culture and sensitivity testing using the standard microbiological procedure. Data is analyzed on SPSS 16.

**Results:**

A total of 184 samples were collected in 4 months. The Male to Female ratio was 1:2 (n=58/126) with mean age 48.5±12 years. 83(45.6%) patients were between 45-60 years. Most common isolated pathogen was Eschericia coli 108(59%) followed by staphylococcus aureus 30(16.4%) and Klebsiella 20(11%). 55(30%) pathogens showed sensitivity to 4-6 antibiotics, 22(12%) strains to 7-9 antibiotics, 33(18%) were sensitive to ≤3 drugs and in 3(1.6%) patients resistance to all antibiotics is seen. The more resistant pathogens were sensitive to intravenous antibiotics alone.

**Conclusion:**

In this low socioeconomic cohort with UTI nearly half the isolated pathogens has shown resistance to most of the commonly used antibiotics recommended in the guidelines especially the floxacin group probably because of its unwarranted use. Therefore, a revised line of management should be developed locally in accordance with the susceptibility pattern of the urinary pathogens to avoid further resistance as well as morbidity of the patient.

## INTRODUCTION

Escherichia Coli is the commonest universal bacterium causing urinary tract infections (UTI) in humans. According to the University of Michigan up to 40% of women will develop UTI at least once during their lives, and a significant number of these women will have recurrent urinary tract infections.^1^

Studies done over the last decade have demonstrated that fluoroquinolones are increasingly being used instead of trimethoprim-sulfamethoxazole (TMP-SMX) to treat UTI’s in ambulatory women, the majority of whom had acute uncomplicated cystitis. Concurrently, and perhaps consequently, fluoroquinolone resistance among uropathogens has increased in prevalence.^2^

This emerging antimicrobial resistance among uropathogens makes the management of acute uncomplicated cystitis increasingly challenging. Several factors had been proved to influence this resistance, lack of hygiene, lack of compliance to dose and duration of the prescribed antibiotic, unjustified prescription writing by quacks and doctors, in addition there is easy access to over the counter medication including antibiotics especially in our part of the region.

Another growing concern is the emergence of beta- lactamase (ESBL) producing gram negative bacteria including E. Coli and Klebsiella species that are multidrug resistant being resistant not only to all the generations of Cephalosporins but also to the fluroquinolones and beta lactam inhibitors/ lactamase inhibitors (Piperacillin/Tazobactam) combinations and therefore leaving only Carbapenem as a sole alternative for therapy in all UTI’s.^3^

This study was aimed to determine the antibiotic sensitivity pattern of the commonest microbial organisms isolated from urinary tract infection. Although a number of studies had been conducted in our country in this respect still continuous monitoring of upcoming resistance to antibiotics has to be addressed at regular intervals in order to modify the guidelines accordingly.

## METHODS

This was a hospital based cross sectional descriptive study. All consecutive patients presenting in the outpatient department of a tertiary care hospital (Creek General Hospital-Korangi), catering a population belonging to low socio economic strata were included as subjects after informed consent. Sampling technique used was non probability convenience. In patients were not included in the study as the collection might be questionable (e.g. in catheterized patients) because of contamination. Duration of study extends to 4 months from December 2014 till March 2015. Adult patients with symptomatic and documented UTI in urine detailed report (pus cells >10) were enrolled. A clean catch midstream urine specimen of approx 50ml was collected in a sterile screw capped, wide mouthed leak proof container for culture and sensitivity testing using the standard microbiological procedure.

Using a calibrated loop method, 10µL of the uncentrifuged specimen was transferred on to the Mckonkey’s agar plate and streak, using the modified Mayo’s technique for identifying lactose and non lactose fermentors and incubated at 35-37ºC for 24 hours. A specimen was considered positive for UTI if a single organism was cultured at a concentration of 10^5^ Colony Forming Units/ml. CLED (Cystein Lactose Electrolyte Deficient) medium is further used for identification and isolation of urinary pathogens.

In the of presence of any potential growth, antibiotic sensitivity testing was done by the Modified Kirby- Bauer disc diffusion method according to the Clinical Laboratory Standards Institute (CLSI) guidelines. The antibiotic strength of 20 antibiotics were observed against the most three frequent UTI pathogens cultured. The antibiotics tested for sensitivity were Gentamicin(30μg), Amikacin(30μg), Imipenem(10μg), Tazobactam(110μg), Septran(25μg), Fosfomycin(50μg), Nitrofurantoin(300μg), Tobramycin(30μg), Amoxacillin/clavulinic acid 2:1(30μg), Cefixime(30μg), Cefuroxime(30μg), Cefradine(30μg), Cefoperazone(75μg), Ceftriaxone(30μg), Cefotaxime(30μg), Ceftazidime(30μg), Ciprofloxacin(5μg), Ofloxacin(5μg), Levofloxacin(5μg) and Moxifloxacin(5μg). Data analyses for the mean ± SD and frequencies with percentage was done on SPSS version 16. Following Operational Definition were used in the present study:

- Microscopic findings of more than 10 WBC per high power field in a urine sample was considered significant for urinary tract infection.
- Significant bacteriuria was defined as culture of a single bacterial species from the urine sample at a concentration of more than 100,000 cfu/ml.^4^

## RESULTS

A total of 200 samples were collected over a period of 4 months with symptomatic and documented urinary tract infection according to the detailed analysis of urine. However 184 patients were positive for urine culture and sensitivity which were then analysed for resistance of the antibiotics tested. Male to Female ratio is 1: 2 (n=58/126). Mean age was 48.5±12 years. 83(45.6%) patients were between 45-60 years and 160 patients were married.

Eschericia Coli(59%) was found to be the commonest microbial agent identified leading to UTI followed by Staphylococcus aureus (16.4%), Klebsiella (11%), Serratia 4%, Enterobactericae 4.3%, Pseudomonas 3% and Streptococcus 2%. In addition, there were some cases reported where more than one organism were found to be the cause of infection. Proteus was found as coinfection in four subjects.

Number (%) of strains of each pathogen sensitive to number of antibiotics is tabulated in Table-I. It was observed that the more resistant pathogens were sensitive to intravenous antibiotics only. We have also observed that 3(1.6%) pathogens had sensitivity to none of the antibiotics tested. The antimicrobial strength of antibiotic agents against the three most frequent pathogens is demonstrated in Table-II.

**Table-I:** Number (%) of strains of each pathogen sensitive to number of antibiotics.

*Organism*	*(1-3)*	*(4-6)*	*(7-9)*	*(>9)*	*None*	*Totaln*
Escheraecia. coli	25	37	16	28	2	108(58.6%)
Klebsiella	4	5	1	9	1	20(11%)
Staphylococcus. Aureus	1	8	5	17	0	31(17%)
Pseudomonas	1	3	1	1	0	6(3%)
Serratia	1	2	2	2	0	7(4%)
Enterobacter	1	0	2	5	0	8(4.3%)
Streptococcus	0	0	2	2	0	4(2%)

**Table-II:** Percentage of sensitivity of each pathogen to the antibiotics.

*Antibiotics used*	*% of E.Coli (n# 108) strains sensitive to antibiotic*	*% of Klebsiella (n# 20) strains sensitive to antibiotic*	*% of S.aureus (n# 31) strains sensitive to antibiotic*
Amikacin	78	70	71
Gentamycin	37	50	52
Tobramycin	32	21	55
Cefuroxime	25	50	71
Cefixime	25	40	16
Cephradine	7	0	47
Cefotaxime	37	47	74
Ceftriaxone	38	45	58
Ceftazidime	34	45	42
Cefoperazone	25	25	62
Fosfomycin	60	53	65
Imipenem	93	85	97
Augmentine	22	45	77
Ciprofloxacin	28	40	39
Levofloxacin	28	54	32
Ofloxacin	26	40	32
Moxifloxacin	27	20	61
Tazobactam	69	70	87
Septran	16	35	26
Nitrofurantoin	59	25	86

## DISCUSSION

Urinary tract infections are the second most common infectious presentation in community. There are an estimated 150 million UTIs per year, worldwide.^5^ Having a predeliction for women it is stated that nearly one woman out of three, will have at least one episode of UTI requiring antimicrobial therapy by the age of 24 years, and almost 50% of all women will experience at least 1 episode of UTI during their lifetime.^6^

The World Health Organization (WHO) describes the results of its first global surveillance report on antibiotic resistance as a ‘cause for high concern. Women have a one-in-three lifetime chance of developing a UTI, which is about 50 times more than for men.^7^

Etiology of UTI shows a diverse group of uropathogens of which the commonest pathogen invovled is E.Coli, a gram –ve facultative anaerobe responsible for 80% of UTI cases in women aged 18-39 years, followed by Staphylococcus saprophyticus and the less common Klebsiella, Enterobacter, Serratia, Proteus, Pseudomonas and Enterococcus.^8^

In comparison, *E.coli*(59%) has also proven to be the most frequent pathogen isolated in our subjects. Females had come twice more frequently with UTI than men in our cohort, a fact which is already established and is also contributed by the anatomical difference from males. *E.Coli* is followed by *Staph.aureus* accounting for 17% and Klebsiella 11% of the urinary pathogens It is also postulated in established data that sexually active females are more prone to develop UTI which is again endorsed by our observation which shows 160 subjects of the total sample(n#184) obtained were married and sexually active.

An important fact to be realized is resistance to antibiotics which has been developing with every new discovery of antibiotics, multiple factors are to be blamed but even in the most developed nations the problem of antibiotic resistance is present, as the pathogens have fought for their own survival, newer mutant strains had developed thus making it more difficult to control the infection. The discovery of newer antibiotics had somewhat taken a slower pace as compared to the emerging lethal strains in the last one and a half decade, despite, of all the advanced researches which gives these pathogens an edge to our species.^8^,^9^ Therefore, a very targeted treatment is necessary for a definite period in these infections to prevent antibiotic resistance as their would no longer be stronger antibiotics left to cater these infections in a few more years. Cost effectiveness of the antibiotics needed to treat these multidrug resistant (MD) and extended multidrug resistant (EMDR) strains is another issue to be dealt with as this would prove to be very expensive.

In this study, E Coli showed the highest sensitivity to imipenem 93% followed by amikacin 78%, tazobactam 69%, fosfomycin 60% and nitrofurantoin 59%, interestingly the floxacin group showed only 26-28% sensitivity, also including was cefixime 25% and augmentine 22%, which explains the failure of response to treatment on empirical basis. Fosfomycin 59% was the other oral antibiotic which has shown some sensitivity.

Klebsiella had also shown similar pattern of sensitivity only the percentages are lower when considering imipenem 85%, amikacin 70% and tazobactam 70%. Response to floxacin group was better than E.coli although not satisfactory, being the most sensitive to Levofloxacin 54% then ciprofloxacin 40%, ofloxacin 40% and only 20% to moxifloxacin. In the cephalosporin group Klebsiella had shown maximum sensitivity to cefuroxime 50%. About 53% were sensitive to fosfomycin and only 25% to nitrofurantoin unlike E.coli.

Staphylococcus Aureus sensitivity to antibiotics was better than E.coli and Klebsiella. Maximum sensitivity was to imipenem 97%, Tazobactam 87%, Nitrofurantoin 86% and Augmentine 77% followed by cefotaxime 74%, Amikacin 71% and cefuroxime 71%. 65% were sensitive to fosfomycin. Most of the subjects were not literate with unsatisfactory hygiene and although the method of collection was explained specifically the possibility of contamination cannot be ruled out.

According to these results, all the first line antibiotics which are recommended in international guidelines for e.g. ciprofloxacin and ofloxacin had shown only 40-50% sensitivity to the commonly prevalent pathogens, similarly co- trimoxazole which was the most commonly prescribed and recommended antibiotic in the international guidelines was only sensitive to 16% of the total E.coli, 35% of Staph aureus and 26% of Klebsiella strain, more importantly a single antibiotic had not shown equally good sensitivity to all three organisms and therefore a 100% response is not expected if the antibiotics were to be given empirically.

Different antibiotics were suggested to overcome the problem of resistance in multiple studies. In this context, according to one study the use of ofloxacin was suggested as the drug of choice for the treatment of UTI caused by either Gram positive or Gram-negative pathogens. This, however, cannot be endorsed as in our study only 28 out of 106 cases were sensitive to ofloxacin and the overall maximum response to floxacin group was only 28%. Gentamicin was additionally recommended for E. coli in the same study.^10^ In contrast, according to our results amikacin would be a better choice since its sensitivity proved better 78% than gentamicin 37%.

The duration of prescribed antibiotics to achieve a favourable outcome also varied in some established data from local studies as compared to what is recommended in international guidelines, as in one study outcome of patient with appropriate antibiotics was higher at 97.3% for five days compared to 83.3% for three days.^11^

Another study conducted in India proved that the hospital acquired E.coli in UTI was more aggressive and difficult to control needing at least one IV antibiotic preferably cephalosporin along with an oral antibiotic when compared with community acquired E.coli, again endorsing the prevalence of resistance in UTI needing inpatient treatment.^12^ Shifali and Gupta in their study done on females proved maximal susceptibility pattern of pathogens predominantly to Amikacin and Nitrofurantoin favouring our results.^4^

An alarming observation in our study was that the most sensitive antibiotics were Imipenem, meropenem, tazobactam, gentamicin and amikacin, all of these antibiotics are given intravenously which requires hospital admission and monitoring, the side effect profile is toxic not to mention the cost that the patients or the government has to bear as these are very expensive.

Resistance to the first line agents in the management of urinary tract infection is not only observed in south Asia including India, Pakistan and Iran but also in the Western as well as European region,^13^ Fluoroquinolone resistance in E. coli in the U.S. had been increasing and current rates of resistance in urinary isolates at UMHS is 27%. In contrast, nitrofurantoin resistance remains less than 5%. Therefore attention should be paid to find out a reason other than the known risk factors that could be held responsible for this resistance.^1^ An observation by Hussain et al. in his study demonstrated ESBL plasmids were readily transferable, which may lead to rapid acquisition of MDR genes by docile strains thus spreading resistance, this finding is very alarming.^14^

Our study has its limitation of not categorically observing the microbial strains responsible for infection in the inpatients and outpatients and diabetics and non diabetics. In addition, history of prior stones, any instrumentation (catheterization etc.) was not taken it would have reflected the predisposing factors also, however these objectives were beyond the scope of our study.

We have used references of different field populations to compare our results. The pattern of resistance can be seen in the last five years in different regions of Pakistan (Table-III), almost all is representing resistance of first line drugs recommended in the guidelines, the question to answer is how then can be an infection effectively treated and at the same time would reduce the chances of resistance. Emperic antibiotic selection is determined in part by local resistance pattern, therefore the local guidelines for treating complicated and uncomplicated UTI was much needed according to the available statistical data in different geographical regions of Pakistan. Following are the recommendations as a result of comparative literature review of the local and international statistics which may benefit the task of reducing resistance in our region.

**Table-III:** Comparative data on antibiotic resistant patterns in local studies over the last five years.

*Settings*	*Sample*	*% of Resistant Strains*	*% of Sensitive Strains*
Lahore-PJMD. 2014.^11^	402 isolates. E.coli(80%), S.aureus(9%), Proteus(5.4%), Pseudomonas(5.2%).	Penicillin (100%), Cefotaxime(89.7%), Ceftazidime (73.8%), Cephradine (73.8%), Augmentin (62.6%), Gentamycin (59.8%), Cefuroxime (58.2%), Ciprofloxacin (54.2%), Ceftriaxone (43.3%), Imipenem (43.3%) 261 (81%) were declared as Multiple drug resistant.5 (1.5%) were Extensive drug resistant.	Streptomycin (70%), Tazocin (86%), Amikacin (87%) and Norfloxacin (89%)
Karachi. Int J Infect Microbiol. 2013.^10^	100 isolates	E.Coli: Amoxacillin (20%), Cefixime (21%), Cefradine (46.5%), Genta (37%), Ofloxa (28%). Kleb: Amoxacillin (61%), Ceftriaxone (77%), Cefradine (73)%, Genta (77%), ofloxacin (93%). Entero: Amox (16%), Cefixime (21%), Ofloxacin (66%), Cefradine (33%), Gentamicin (100%).	Imipenm (100%) Imipenem (77%) Imipenem (100%)
Pak J Zool. 2012^17^	E.Coli: 310 isolates	Sensitivity: 20%< 5 drugs 14% 5-8 drugs Cipro 57%, ofloxacin 57%, cephradine 60%, septran 87%, norfloxacin62%	Resistance: 65% >8 drugs Imipenem 100%, mero 100%, tazo/pip 97%, amikacin 90%, cef/sulbactam 87%, ceftazi 77%, gentamicin 73%
PIMS. 2010^18^	100 isolates E.coli. 60%, Pseudo7%, klebsiella13%, S. aureus10%, Enter2%	Amoxacillin (85%), Augmentine (85%), Ciprofloxacin (65%), Levofloxacin (47%), Cefradine (45%), Nitrofurantoin (73%).	Cefoperazone (54%), Ceftriaxone (50%), Amikacin (76%), Imipenem (91%), Pipracillin/Tazobactam (91%),
This study: 2016 Karachi	184 isolates *E.coli.*59%, *S. aureus*17%, Klebsiella11%, Pseudo3%, Enter4%, Serratia4%, Streptococcus2%	E.Coli:Cipro 72%, Ofloxacin74%, Levo 72%, Aug 78%, Septran 84%, Cefurox 75%, Ceftriaxone 62%, Cefixime 75%. S.aureus: Cipro 61%, Ofloxacin 68%, Septran74%, Cifixime 84%. Klebsiella:Cipro 60%, Oflox 60%, Cifixime 60%, Aug 55%, Septran 65%, Nitrofurantoin 75%.	E.Coli: Imipenem 93%, Amikacin 78%, Tazo 69%, Fosfomycin 60%, Nitrofurantoin 59%. S.Aureus: Imipenem 97%, Tazo 87%, Nitrofurantoin 86%, Aug 77%, Cefotax 745, Amikacin 71%, Ceftriaxone 58%, Klebsiella:Imipenem 85%, Tazo 70%, Amikacin 70%

1. We suggest that empirical antibiotic selection should be based on the knowledge of local prevalence of bacterial organisms and antibiotic sensitivities rather than on international guidelines.
2. Culture and sensitivity should be done where appropriate.
3. Floroquinolone use should be reserved because this is the only orally active drug which works against Pseudomonas and other multidrug resistant bacteria,^15^,^16^ as in most published data from our region the resistance is in >50% of the cases in hospital setting, this would help in taking the resistance pressure off from the much used quinolone class.^9^
4. Use of nitrofurantoin and fosfomycin should be incorporated in our guidelines as they have better efficacy as well as low impact on promoting antibiotic resistance. In addition, Nitrofurantoin had also been approved in pregnancy because of its fewer adverse reaction.^15^

## CONCLUSION

Emergence of multidrug resistant organisms poses a great public and therapeutic threat to clinicians all over the world but especially in our geographical region. The problem can only be catered effectively with formulation and strict implementation of a local therapeutic guidelines in accordance with the susceptibility pattern of pathogens existing in our own community. In addition, continued surveillance and monitoring of antimicrobial resistance would help in improvising our line of management effectively.

### Authors’ Contribution

***Saera Suhail Kidwai:*** Conception and design, acquisition of data, Critical revision of article, and final approval of the article. ***Ayesha Nageen:*** Contributed to conception and design, Analysis and interpretation of data, drafting the article. ***Samina Ghaznavi:*** Interpretation of data, drafting the article, final approval of the version to be published. ***Farhat Basheer:*** Interpretation of data, drafting the article, final approval of the manuscript. ***Jamal Ara:*** Conception, design, final critical analysis and approval of manuscript.
